# Impact of nursery-to-fattening dietary transitions on gut microbiome composition and growth performance in black soldier fly larvae

**DOI:** 10.3389/fmicb.2026.1901348

**Published:** 2026-08-19

**Authors:** Kato Cerckel, Lotte Frooninckx, Sabine Van Miert, Jeroen De Smet, Freek IJdema

**Affiliations:** 1 Research Group for Insect Production and Processing, Department of Microbial and Molecular Systems (M^2^S), KU Leuven, Geel, Belgium; 2 Centre of Expertise Sustainable Biomass and Chemistry, Thomas More University of Applied Sciences, Geel, Belgium

**Keywords:** bacterial communities, bacterial community plasticity, diet shift, growth performances, *Hermetia illucens*, rearing systems

## Abstract

Black soldier fly (BSF) (*Hermetia illucens*) larvae are used in large-scale bioconversion due to their capacity to convert diverse organic substrates into high-quality biomass. To improve circularity, rearing practices must be optimized, especially when nutritionally poor, low-cost substrates are used. Production systems often adopt a two-phase feeding strategy in which larvae first receive a nutrient-rich nursery diet before transitioning to a fattening diet. The effects of such a diet shift on the gut microbiome and larval performance remain unclear. This study explores how a diet shift between the nursery stage (0–7 days after egg harvest; DAH) and the fattening stage (DAH 8–15) influences larval performance and microbiome composition. Chicken feed (CF) and artificial supermarket food waste (SFW) were used as contrasting diets across four conditions: continuous feeding (CF to CF and SFW to SFW) and diet shifts (CF to SFW and SFW to CF). At the end of the nursery phase, there was no significant difference in larval weight between the two diets. Immediately after the diet shift (DAH 8–9), CF-nursed larvae were heavier than SFW-nursed larvae, but SFW-nursed larvae gradually reached comparable weights over time. This convergence occurred more rapidly when larvae were maintained on SFW. Notably, survival within both fattening diets was lower for SFW-nursed larvae than for CF-nursed larvae, which may have reduced larval density and feed competition during the fattening phase. Microbiome profiling through 16S rRNA gene amplicon sequencing revealed distinct bacterial communities associated with CF- and SFW-based rearing: CF-reared larvae maintained a consistently diverse community, whereas SFW-reared larvae exhibited low diversity. Following a diet transition, microbial composition shifted toward a fattening-driven profile. However, the rate and extent of this shift depended on the fattening substrate. Under SFW fattening, microbiome differences between nursery treatments disappeared by the end of the experiment, whereas several biomarkers persisted under CF fattening, suggesting a stronger legacy effect of the nursery diet. Overall, these findings demonstrate that interactions between diet, microbiome, and larval physiology shape performance in two-phase rearing systems, highlighting that early-life nutrition can influence microbiome trajectories and affect responses to subsequent feeds, an important consideration for optimizing BSFL production.

## 1 Introduction

Insects are recognized as a valuable alternative feed source for livestock, as they can provide high-quality nutrients while simultaneously valorizing organic waste streams. Among these, the black soldier fly larvae (BSFL) (*Hermetia illucens*, Diptera; Stratiomyidae) have shown particular potential for commercial production due to their high nutritional value, including a crude protein content (~35–50% DM), a substantial lipid content (~20–35% DM), and a well-balanced amino acid profile, as well as their ability to convert diverse organic side streams into insect biomass (Chia et al., 2020; Sheppard et al., 1994; Li et al., 2022; Liu et al., 2017). As in traditional livestock systems, carefully designed rearing strategies are essential for ensuring consistent larval development and producing high-quality biomass. This includes the management of environmental conditions such as temperature and relative humidity, and the optimization of larval density and diet (Harnden and Tomberlin, 2016; Holmes et al., 2012; Klammsteiner et al., 2025; Ribeiro et al., 2022). Although the use of organic waste streams enhances the sustainability of BSFL farming, such substrates often vary in their nutritional and/or physiochemical composition and may not reliably supply all nutrients required for optimal growth and performance (Broeckx et al., 2021). This situation introduces competing objectives in insect farming. Applying diet optimization strategies derived from conventional livestock systems supports consistent larval growth and biomass quality. Conversely, this may constrain the effective incorporation of organic waste streams, which is one of the principal benefits of insect bioconversion.

The trade-off between diet optimization and organic waste use raises the question of which production stage is most critical to optimize. The BSFL rearing substrate requirements vary across the production stages, which are typically divided into two consecutive stages (1) the nursery stage and (2) the fattening stage (Barrett et al., 2023; Grassauer et al., 2023). During the nursery stage, larvae are highly sensitive to non-standardized diets, because their small size makes excess or poorly digested feed prone to mold formation, which restricts movement and can cause mortality. Therefore, high-quality, standardized diets are typically provided to support survival and stable early development (Grassauer et al., 2023). In the subsequent fattening stage, the larger larvae are better adapted to process a wider range of organic substrates, as long as these provide sufficient nutritional value to support further development (Grassauer et al., 2023). Because waste- and side streams are typically introduced during the fattening stage, many BSFL nutrition studies initiate experiments with larvae that are already several days old (e.g., 4–7 days old) (Broeckx et al., 2021; Frooninckx et al., 2024; Li et al., 2022; Vandeweyer et al., 2023). As a result, the experimental focus is generally on the fattening stage, while the nursery stage has received comparatively less attention (Broeckx et al., 2021; Frooninckx et al., 2024; Li et al., 2022; Vandeweyer et al., 2023). The industrial implementation of these stages differs across systems, with centralized facilities completing both stages in the same location, while decentralized systems rear larvae at one site during the nursery stage before moving them to another site for fattening (Grau et al., 2022). In both approaches, dietary shifts can occur due to the fattening stage taking place at a different location and/or the use of lower-quality, cost-effective substrates during this stage. Such shifts may influence larval performance, including growth, development, and survival.

Aside from providing the nutrients essential for larval growth and development, larval diet also modulates the gut microbiome, either by introducing new substrate-borne microorganisms or by providing resources that promote the growth of specific taxa within the existing community (Bruno et al., 2019; Chen et al., 2023). The gut microbiome can provide benefits for its insect host through nutritional functions, as well as protection against pathogens or detoxification of volatile components in the diet (Chabanol and Gendrin, 2024; Shamjana et al., 2024). Moreover, microbial colonization is considered important for larval development, as they are known to contribute to key functions such as aiding nitrogen acquisition, synthesizing vitamins, and metabolizing lipids and carbohydrates in various insect species (Breznak et al., 1973; Chabanol and Gendrin, 2024). Li et al. (2023) demonstrated these functions in BSF using germ-free larvae, where gnotobiotic larvae supplemented with *Citrobacter*, *Dysgonomonas*, *Klebsiella*, *Ochrobactrum*, or *Providencia* exhibited increased larval weight compared to axenic larvae, highlighting the potential role of specific gut bacteria in promoting growth and metabolic efficiency. However, these findings were obtained under gnotobiotic conditions and may not fully reflect conventionally reared larvae. Importantly, a disruption of the larval microbiome composition, known as dysbiosis, can impair these functions and potentially cause harm to the insect or inhibit its growth (Chabanol and Gendrin, 2024; Shamjana et al., 2024). Gut dysbiosis can result from factors such as sudden dietary changes, antibiotic use, or environmental stresses such as heat stress (Gresse et al., 2017; Ringseis and Eder, 2022; Silvestro et al., 2025). In traditional livestock, abrupt changes in diet have been shown to disrupt the gut microbiome, causing diarrhea and other health disorders (Gresse et al., 2017; Khalil et al., 2022; Wang et al., 2023; Welch et al., 2022). In BSFL, gut dysbiosis has been observed under both low-nutrient diets (Ramona et al., 2022) and diets with a high protein content relative to other macronutrients, which is hypothesized to reduce larval performance (Bruno et al., 2019). These findings indicate that dietary transitions represent a critical vulnerability in livestock production and may similarly affect BSFL performance. However, most studies explore specific, single diets during the fattening stage and their effect on larval production, omitting the importance of diet choices during the nursery stage. Therefore, the effects of dietary shifts on BSFL performance and their bacterial composition remain poorly understood.

The aim of this study was to investigate diet shifts between the nursery and fattening stages and assess their impact on larval performance and gut microbiome dynamics. To achieve this, larvae were nursed on either chicken feed or an artificial supermarket food waste diet formulated to mimic food waste. In the fattening stage, they were either switched to the alternative diet or remained on their original diet. Changes in bacterial community compositions and larval performance were monitored throughout the experiment to evaluate the effect of diet shifts.

## 2 Materials and methods

### 2.1 Diet preparation

Two different diets were used in this study. The first diet consisted of chicken feed (CF, Chicken Start Mash 259, AVEVE, Belgium), which is a standard diet used in BSFL rearing. The CF was finely ground for application during the nursery stage and coarsely ground for use during the fattening stage. CF was freshly prepared and mixed with tap water prior to use to ensure consistency in composition and achieve the target moisture content. The second diet consisted of an artificial supermarket food waste (SFW) designed to mimic supermarket food waste, based on the recipe by Lievens et al. (2022), and was prepared by blending (Robot Coupe Blixer 23 and Blixer 5 v.v.) the following ingredients: tomatoes (12.1 wt%), cheese (1.5 wt%), yogurt (0.5 wt%), apple (12.1 wt%), bread (22.2 wt%), potatoes (8.8 wt%), iceberg lettuce (13.0 wt%), kidney beans (5.9 wt%), cucumber (11.9 wt%), and zucchini (12.0 wt%). SFW was stored at −20 °C and thawed in a refrigerator prior to use to avoid any cold-induced effects on its properties. The nutritional composition of CF has been described by Broeckx et al. (2021), while the nutritional composition of SFW was calculated based on the quantities and nutritional profiles of its ingredients (Table 1).

**Table 1:** Nutritional composition and WHC of chicken feed (CF) and artificial supermarket food waste (SFW): with dry matter content (DM), ether extract (EE), crude protein content (CP), carbohydrates: amylase-treated neutral detergent fiber content (aNDF) and non-fiber carbohydrate content (NFC), gross energy content (GE), and water holding capacity (WHC).

Substrate	DM ^**1**^	CP ^**2**^	EE ^**2**^	Carbohydrates ^**2**^		GE ^**3**^	WHC (%)
				aNDF ^**2**^	NFC ^**2**^		
CF	89.63 ± 0.22^a^	20.4 ± 2.4^b^	4.6 ± 0.4^b^	20.9 ± 1.2^b^	48.2 ± 5.3^b^	315.0^b^	114.86 ± 7.31^a^
SFW	22.19 ± 0.12^a^	14.1^c^	4.9^c^	73.7^c^		388.4^c^	-41.08 ± 1.33^a^

1 g/100 g fresh matter, ^2^ g/100 g dry matter, ^3^ kcal/100g dry matter, ^4^ g water retained/ 100 g fresh matter, ^a^ Measured in this study, ^b^ Broeckx et al., 2021, ^c^ Calculated based on quantities and nutritional profiles of ingredients.

For both substrates, the dry matter (DM) content and water-holding capacity (WHC) were measured (Table 1). DM was determined gravimetrically by weighing 5 g of sample before and after drying at 105 °C for 24 h in a forced-air oven (Practum 224–1 s, Sartorium). The WHC, representing the maximum water content a substrate can retain when fully saturated (Koviessen et al., 2023), was determined based on the method described by Frooninckx et al. (2024). Briefly, 5 g of substrate and 20 mL of water were added to a 50 mL Falcon tube, followed by vortexing. After one hour of absorption time, the mixture was centrifuged at 10000 × g and 20 °C for 30 min. The supernatant was decanted, and the tube was inverted for 30 min on absorbent paper to remove residual free water. WHC was calculated as:


$$\mathrm{WHC}\;(\%)=\frac{m_{3}-m_{1}}{m_{2}}$$

m_1_ (g) = weight of the falcon tube + dry sample.
m_2_ (g) = weight of the dry sample.
m_3_ (g) = weight of the falcon tube + sample after centrifugation.

Negative WHC values indicate the presence of free water, meaning that water is present in excess of the substrate’s retention capacity. A WHC value of zero corresponds to saturation, while positive values indicate remaining water-holding capacity within the substrate.

### 2.2 Experimental setup

Larvae were reared in two consecutive stages, the nursery and the fattening stage. Days after egg harvest (DAH) were used to indicate timing throughout the experiment, with DAH 0 corresponding to the day the eggs were harvested, at which point the eggs were between 0 and 48 h old.

The experimental design was adapted from the rearing methodology described by Frooninckx et al. (2024). To accommodate the small-scale nature of the present study, the quantities of eggs and substrate were proportionally reduced while maintaining the same egg-to-substrate ratio on a wet weight basis, feeding regime, and general rearing procedures as described in the original protocol.

At the start of the nursery stage (DAH 0), 0.2 g of BSF eggs were weighed and placed on a plastic weighing dish positioned on the CF or SFW nursery substrates in 300 mL cups (diameter 9.5 cm, height 10.5 cm; AVA). The amounts and ingredient composition of the substrates used at each experimental stage (Table 2) were standardized, and all diet treatments were supplied with an equal total weight of diet. During the nursery stage, diets were supplied at DAH 0 and 4, whereas in the fattening stage, substrate was provided only once, at DAH 7 in an amount sufficient for the remainder of the experiment.

**Table 2:** Substrate formulations of chicken feed (CF) and artificial supermarket food waste (SFW) provided during the nursery stage (at DAH 0 and 4) and fattening stage (at DAH 7).

Substrate	Stage	DAH	Weight of the ingredients		
			CF (g)	Water (g)	SFW (g)
CF	Nursery	0	20.00	30.00	-
		4	48.00	72.00	-
	Fattening	7	99.00	120.00	-
SFW	Nursery	0	-	-	50.00
		4	-	-	120.00
	Fattening	7	-	-	219.00

On DAH 2 and 3, each substrate was mixed using a spoon, and the egg-containing weighing dish was removed on DAH 3. On DAH 4, larvae along with their substrate were transferred to larger transparent 1 L cups (diameter 11.5 cm, height 13.5 cm; AVA), with additional substrate added as needed to meet their nutritional requirements. From this stage, a layer of aluminum foil was placed over the substrate to reduce water loss through evaporation.

On DAH 7, 170 larvae per cup were transferred into 1 L cups containing one of the two fattening substrates covered with a layer of aluminum foil, and the fattening stage continued until DAH 15. The number of larvae was selected to maintain a manageable small-scale experimental setup while ensuring sufficient individuals for sampling and reliable determination of survival rates. This setup resulted in four experimental conditions, covering all combinations of the two nursery substrates and two fattening substrates. A total of six trials were conducted. Each experimental condition was repeated a total of six times across these trials (Supplementary Table 1). All rearing experiments were carried out at 27 °C and 60% relative humidity to ensure standardized rearing conditions across treatments.

### 2.3 Performance metrics in insect rearing

Individual larval weight was determined by weighing larvae at specific time points using an analytical balance (Sartorius Practum 224–1S). At DAH 7, five groups of ten larvae were weighed. At DAH 8, 9, 10, 14, and 15, three groups of ten larvae were weighed. The mean individual larval weight was calculated by dividing the total group weight by the number of larvae.

At 15 DAH, final larval performance was evaluated. Survival at the end of the fattening phase was determined by counting the remaining larvae. As 170 larvae were transferred from the nursery to the fattening substrate and three groups of 10 larvae were sampled for microbiome analysis during the experiment (Section 2.4), a maximum of 140 larvae could remain in each cup at DAH 15. Survival rate was therefore calculated using the following formula:

Survival rate(%) $=\frac{\;\text{number of larvae}\;\mathrm{at}\;\mathrm{DAH}\;15}{140}*100$

Subsequently, total larval weight was determined by weighing all surviving larvae collectively using an analytical balance (Sartorius Practum 224–1S), thereby accounting for both the number of surviving larvae and the weight of individual larvae.

For measurements at DAH 8, 9, 10, 14, and 15, performance metrics were normalized per trial to prevent trials with more biological replicates from disproportionately affecting the analysis. Values recorded at DAH 7 were not normalized because no more than one biological replicate was available per nursery diet within each trial. The normalization procedure was applied to all performance metrics, including individual larval weight, survival rate, and total larval weight, according to the following formula:


$$\begin{array}{l} {\bar{X}}_{\mathrm{per}\;\mathrm{DAH},\;\text{conditon},\;\text{biological repeat}}\\ =\frac{\;X_{\mathrm{Per}\;\mathrm{DAH},\;\text{conditon},\text{trial},\;\text{biological repeat}}\;}{{\bar{X}}_{\mathrm{per}\;\mathrm{DAH},\;\text{conditon},\text{trial}}}\\ {}*{\bar{X}}_{\mathrm{per}\;\mathrm{DAH},\text{conditon}} \end{array}$$

$\bar{X}$ _per DAH,conditon,biological repeat_ = normalized value of the performance metric per DAH, per condition, and per biological repeat.
$X_{\mathrm{Per}\;\mathrm{DAH},\;\text{conditon},\text{trial},\;\text{biological repeat}}$ = observed value of the performance metric per DAH, per condition, per trial and per biological repeat.
$\bar{X}$ _per DAH,conditon,trial_ *=* average value of the performance metric per DAH, per condition, and per trial.
$\bar{X}$ _per DAH,conditon_ = average value of the performance metric per DAH, and per condition.
X represents the performance metric under consideration (individual larval weight, survival rate, or total larval weight).

For DAH 8, 9, 10, 14, and 15, the normalized values were used for all subsequent analyses. Normalized individual larval weight values were used to fit growth curves for each fattening substrate to enable comparison of larval weight across nursery substrates. In addition, normalized survival rate and total larval weight values at DAH 15 were used to evaluate final larval performance.

### 2.4 Gut bacterial community analysis

Larvae were collected at DAH 7, 8, 9, 10, and 15. At DAH 7, 50 larvae were sampled to ensure sufficient material for DNA extraction, while at DAH 8, 9, 10, and 15, 10 larvae were collected per time point. All larvae were frozen at −20 °C and surface-sterilized twice with 70% ethanol, followed by a single rinse with Milli-Q water. DNA extraction was performed on samples from DAH 7, 9, 10, and 15. For homogenization, all 50 larvae collected at DAH 7 were pooled and crushed, while at the other selected time points, ten larvae per DAH were crushed. From each homogenate, 100–200 mg was used for DNA extraction using the E.Z.N.A.^®^ Soil DNA Kit, following the manufacturer’s instructions.

DNA samples were sent to Novogene for 16S rRNA gene sequencing, where PCR amplification of the V4 region was performed using primers 515F and 806R following the analytical pipeline previously described by Van Looveren et al. (2024). In brief, paired-end reads were merged using USEARCH (v11.0.667) and merged sequences were truncated at the 250th base. A quality filtering based on recalculated Phred scores was done, where reads under 250 bp or exceeding a total expected error threshold of 0.5 were removed. Mitochondrial, chloroplast, and non-target sequences were removed using Mothur (v1.48.0) with the 16S ITGDB database as the reference (Hsieh et al., 2022). The remaining sequences were clustered into zero-radius operational taxonomic units (zOTUs) by the UNOISE3 algorithm (Edgar and Flyvbjerg, 2015), and the taxonomy was determined by the SINTAX algorithm based in the 16S ITGDB database and considered reliable when bootstrap confidence values exceeded 0.80. All subsequent data processing was executed in R (version 4.3.0). Sequences were filtered by removing reads that had a relative abundance of less than 0.1% per sample. The filtered reads were used to evaluate how the microbial communities responded to the diet shifts. Alpha-diversity indices were calculated and visualized to describe patterns in bacterial richness and diversity across treatments using the *microbiome* package. Given the limited replication and unbalanced distribution of samples across experimental trials, no formal statistical comparisons were performed on these metrics. Statistical inference was instead based on beta-diversity and differential abundance analyses. The effects of diet treatment, trial, and their interaction on beta-diversity estimates based on Hellinger-transformed Bray-Curtis distances were analyzed using a permutational multivariate analysis of variance (PERMANOVA). Beta-diversity was visualized in a Redundancy Analysis (RDA), using the phyloseq and MicroViz packages. The relative abundance of zOTUs was determined per replicate and the mean larval gut bacterial community at each time point and for each treatment was visualized using bar plots. Differences in taxonomic composition among treatments were assessed using DESeq2 using the similarly named R-package. To account for the many zero counts found in amplicon datasets, the reads were normalized using DESeq2’s *poscounts* method of size factor estimation. For each DAH subgroup and treatment comparison, a DESeq2 dataset was constructed using the design formula *~ trial + treatment* (correcting for trial differences and where treatment consists of the different combinations of nursery and fattening diet), and a differential abundance analysis was conducted using the Wald test with parametric dispersion estimation. Results were visualized in volcano plots, with taxa considered significantly differentially abundant when showing an adjusted *p*-value < 0.05 after Benjamini-Hochberg correction and a log2foldchange higher than 0.5.

### 2.5 Statistical analysis

Statistical analyses were performed in JMP Pro 17 using a significance level of *α* = 0.05. Mean individual larval weight was compared between nursery substrates at DAH 7 and between nursery substrates within each fattening substrate at DAH 8, 9, 10 and 15. At DAH 15, individual larval weight, survival rate, and total larval weight were compared between nursery substrates within each fattening substrate and between fattening substrates within each nursery substrate. Normality of the data was evaluated using the Shapiro–Wilk test, and homogeneity of variances was examined using Levene’s test. When both assumptions were met, a parametric Student’s *t*-test was applied. If variances were unequal, Welch’s *t*-test was used. When the normality assumption was not satisfied, comparisons were made using the non-parametric Wilcoxon rank-sum test.

## 3 Results

### 3.1 Substrate characterization

To examine how substrate characteristics might relate to variation in larval growth and microbiome composition, the nutritional composition and WHC of the diets were analyzed. The two substrates differed strongly in their physical properties. When expressed on a DM basis, CF was characterized by a substantially higher DM content (89.63 ± 0.22% in CF, 22.19 ± 0.12% in SFW) and a greater WHC (114.86 ± 7.31 for CF, −41.08 ± 1.33% for SFW) compared to SFW. In addition, CF contained more protein than SFW, whereas SFW contained more carbohydrates than CF (Table 1). Gross energy was also higher for SFW than for CF (Table 1). However, because no distinction was made between fiber and non-fiber carbohydrates in SFW, the GE value is likely overestimated, as fiber contributes to gross energy despite its relatively low digestibility. On an as fed basis, CF also showed a greater WHC than SFW, with free water clearly visible in the SFW substrate (Table 3). In terms of nutritional composition on an as fed basis, the CF diet contained more than twice as much protein and over 1.5 times more fat and carbohydrates compared with SFW (Table 3).

**Table 3:** Nutritional properties and WHC of the substrates chicken feed (CF) and artificial supermarket food waste (SFW) as fed per container during the nursery stage (DAH 0 and 4) and fattening stage (DAH 7). Including dry matter content (DM), crude protein content (CP), ether extract content (EE), carbohydrates: amylase-treated neutral detergent fiber content (aNDF) and non-fiber carbohydrate content (NFC), gross energy (GE) and water holding capacity (WHC).

Substrate as fed	Stage	DAH	DM ^**1**^	CP ^**2**^	EE ^**2**^	Carbohydrates ^**2**^		GE ^**3**^	WHC (%) ^**4**^
						aNDF ^**2**^	NFC ^**2**^		
CF	Nursery	0	35.85	3.66	0.82	3.75	8.64	56.46	-14.05
		4	35.85	8.78	1.98	8.99	20.74	135.51	-14.05
	Fattening	7	40.52	18.10	4.08	18.54	42.77	279.50	-2.87
SFW	Nursery	0	22.19	1.56	0.54	8.18		47.35	-41.08
		4	22.19	3.75	1.30	19.63		113.63	-41.08
	Fattening	7	22.19	6.85	2.38	35.82		207.38	-41.08

1 g/100 g as fed substrate, ^2^ g, ^3^ kcal, ^4^ g water retained/ 100 g as fed substrate.

### 3.2 Larval performances

Mean individual larval weight was measured on DAH 7, 8, 9, 10, and 15. At the end of the nursery stage and the start of the fattening stage, larvae reared on CF reached a mean body weight of 0.0054 ± 0.0009 g, whereas larvae nursed on SFW had a mean body weight of 0.0035 ± 0.0009 g. No significant difference was detected between treatment (Wilcoxon rank-sum test: Z = −1.59, *p* = 0.112). However, the limited number of replicates (*n* = 4 for CF and *n* = 3 for SFW) may have reduced the statistical power of the analysis. Although not statistically significant, the observed difference in mean body weight suggests a trend toward higher initial weights in CF-nursed larvae.

**Figure 1:**
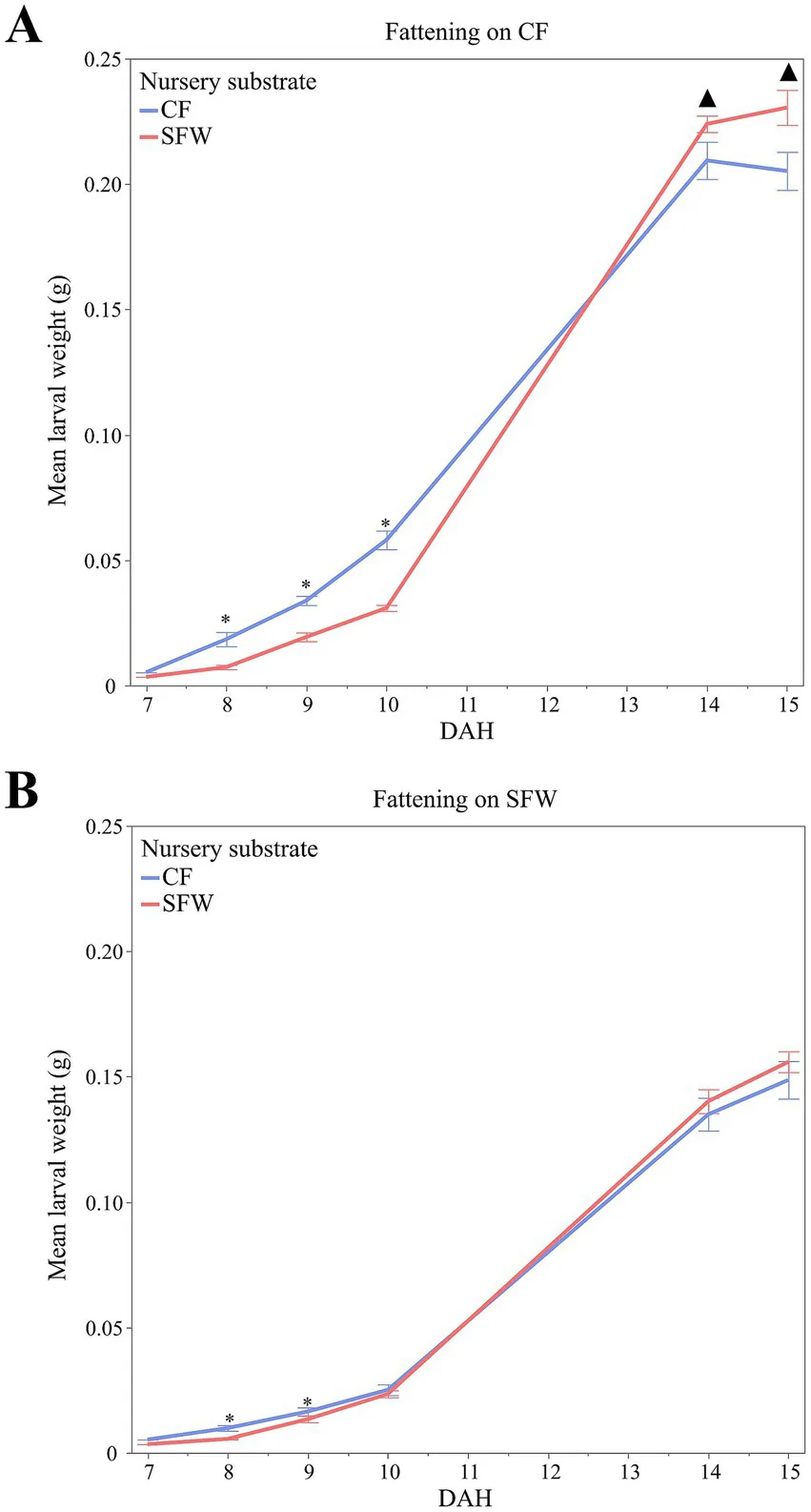
Mean larval weight (g) (± SD) of black soldier fly larvae as a function of days after harvest (DAH), nursed on different nursery diets (chicken feed (CF) or artificial supermarket food waste (SFW)) and fattened on **(A)** CF and **(B)** SFW. At all measuring points, *n* = 6 for each diet-shift group, representing all combinations of the nursery and fattening diets (CF and SFW), except at DAH 7, where *n* = 4 and *n* = 3 for the CF and SFW nursery treatments, respectively. No measurements were carried out on DAH 11, 12, and 13. The asterisk (*) indicates that larvae nursed on CF were significantly heavier than larvae nursed on SFW (*p* < 0.05). The triangle (▲) indicates that larvae nursed on SFW were significantly heavier than larvae nursed on CF (*p* < 0.05).

For larvae fattened on CF, individual larval weight at DAH 8, 9, and 10 was significantly higher for CF-nursed larvae than for SFW-nursed larvae (Figure 1A). At DAH 8, CF-nursed larvae reached a weight of 0.0185 ± 0.0028 g compared with 0.0074 ± 0.0009 g for SFW-nursed larvae (*p* < 0.001). This difference persisted at DAH 9, with weights of 0.0339 ± 0.0018 g and 0.0194 ± 0.0017 g for CF- and SFW-nursed larvae, respectively (*p* < 0.001), and at DAH 10, with weights of 0.0581 ± 0.0037 g and 0.0310 ± 0.0012 g, respectively (*p* < 0.001). However, this pattern shifted in the final stages of the experiment. At both DAH 14 and DAH 15, SFW-nursed larvae reached slightly higher weights than CF-nursed larvae (0.2240 ± 0.0033 g vs. 0.2094 ± 0.0074 g, *p* < 0.001 at DAH 14; 0.2305 ± 0.0070 g vs. 0.2052 ± 0.0076 g, *p* < 0.001 at DAH 15). Notably, CF-nursed larvae showed a decrease in body weight between DAH 14 and 15. While this pattern is consistent with the onset of prepupal development, this cannot be confirmed as developmental stage was not assessed morphologically. Survival at DAH 15 within the CF fattening treatment was significantly affected by nursery substrate, with CF-nursed larvae exhibiting higher survival than SFW-nursed larvae (92.86 ± 3.40% vs. 83.81 ± 3.87%, *p* = 0.0016). Despite differences in individual larval weight and survival, total larval weight at DAH 15 did not differ significantly between nursery treatments, reaching 25.94 ± 0.09 g and 25.93 ± 0.90 g for CF- and SFW-nursed larvae, respectively (Table 4).

**Table 4:** Mean (±SD) values of final performance parameters on different diet combinations at DAH 15, including individual larval weight (g), survival rate (%), and final larval weight (g). Larvae were nursed on either chicken feed (CF) or artificial supermarket food waste (SFW) and subsequently fattened on either CF or SFW.

Nursery substrate	Fattening substrate	Individual larval weight (g)	Survival rate (%)	Final larval weight (g)
CF	CF	0.2052 ± 0.0076	92.86 ± 3.40	25.94 ± 0.09
SFW	CF	0.2305 ± 0.0070	83.81 ± 3.87	25.93 ± 0.90
CF	SFW	0.1487 ± 0.0075	86.79 ± 1.97	16.98 ± 1.05
SFW	SFW	0.1560 ± 0.0041	75.12 ± 3.47	14.98 ± 0.64

A similar pattern was observed for larvae fattened on SFW (Figure 1B). During the first 48 h after transfer, CF-nursed larvae reached significantly higher individual weights than SFW-nursed larvae, both at DAH 8 (0.0099 ± 0.0011 g vs. 0.0056 ± 0.0002 g, *p* < 0.001) and DAH 9 (0.0165 ± 0.0016 g vs. 0.0135 ± 0.0013 g, *p* = 0.004). However, no significant effect of the nursery substrate on individual larval weight was detected from DAH 10 onwards. Survival was significantly higher in CF-nursed larvae than in SFW-nursed larvae (86.79 ± 1.97% vs. 75.12 ± 3.47%, *p* < 0.001). In contrast to the CF fattening treatment, total larval weight at DAH 15 was significantly affected by nursery substrate, with CF-nursed larvae reaching a higher total weight than SFW-nursed larvae (16.98 ± 1.05 g vs. 14.98 ± 0.64 g, *p* = 0.003).

Overall, within both nursery substrates, CF fattening resulted in significantly higher individual larval weight, survival, and total larval weight at DAH 15 than SFW fattening (Table 4; Figure 1). As these comparisons were made at a fixed sampling time point, they may not reflect equivalent developmental stages. The decrease in body weight observed in larvae nursed and fattened on CF suggests that these larvae may have been developmentally more advanced than larvae fattened on SFW, although this could not be confirmed morphologically.

### 3.3 Bacterial community

**Figure 2:**
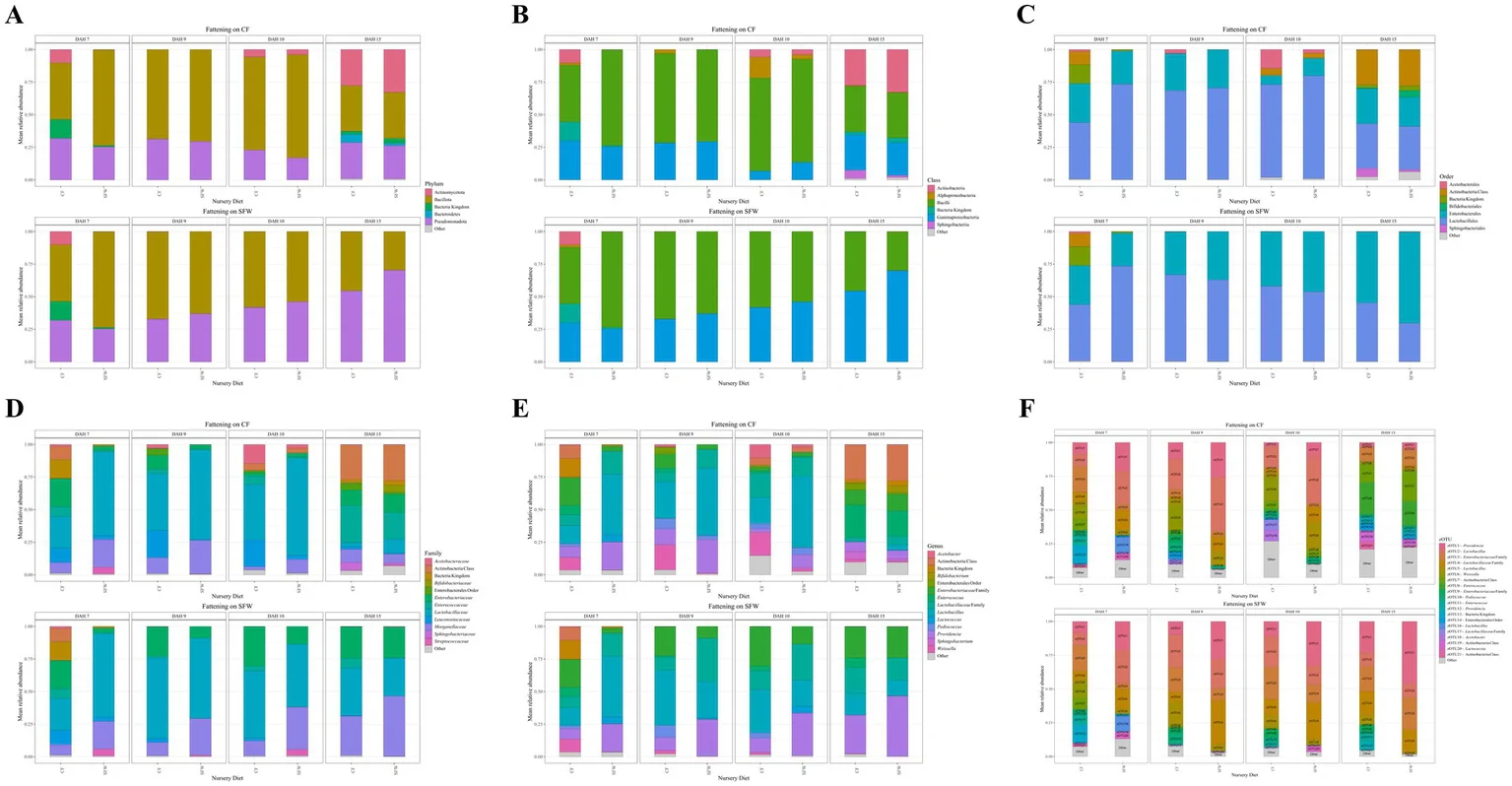
Mean relative abundance of bacterial taxa in the gut microbiome of black soldier fly larvae at the **(A)** phylum, **(B)** class, **(C)** order, **(D)** family, **(E)** genus, and **(F)** zOTU levels at days after harvest (DAH) 7, 9, 10 and 15. Larvae were nursed on chicken feed (CF) or artificial supermarket food waste (SFW) and subsequently fattened on CF or SFW.

**Figure 3:**
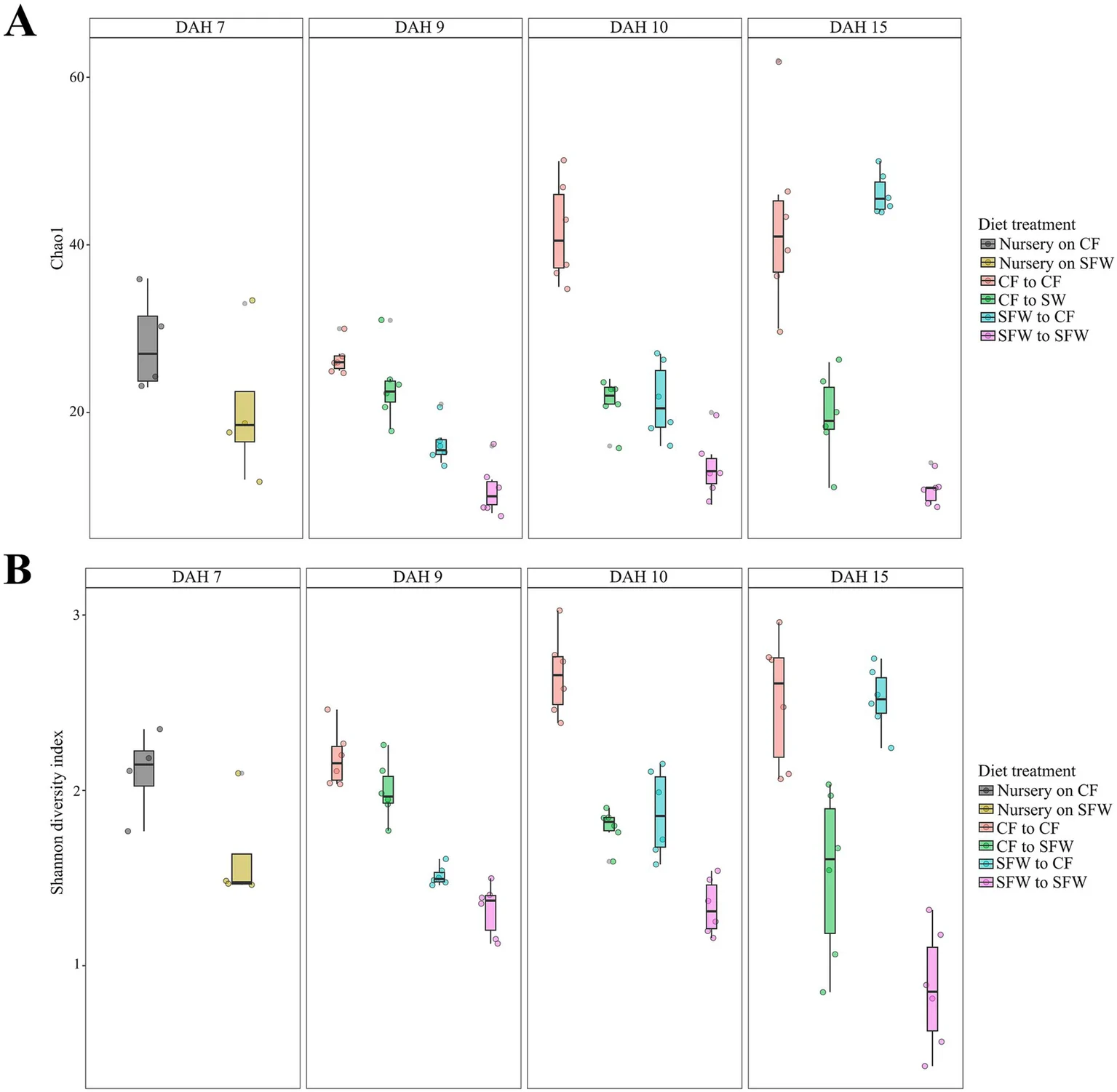
Alpha diversity of black soldier fly larvae reared on different diet combinations at days after harvest (DAH) 7, 9, 10, and 15. **(A)** Chao1-index and **(B)** Shannon diversity index, visualized using boxplots. The central line within each box represents the median. The box boundaries indicate the first and third quartiles (25th and 75th percentiles), corresponding to the interquartile range (IQR). Whiskers extend to the most extreme data points within 1.5 × IQR from the box. Individual points represent biological replicate samples. At DAH 7, larvae were nursed on either chicken feed (CF) (*n* = 4) or artificial supermarket food waste (SFW) (*n* = 3), and at DAH 9, 10, and 15 the diet-shift groups represent all combinations of nursery and fattening diets, namely CF and SFW (*n* = 6).

An overview of the number of reads, taxonomic classification, and sample information of the zOTUs identified through 16S rRNA sequencing is provided in Supplementary Table 2. Bacterial community composition differed markedly between treatments across all taxonomic levels (phylum, class, order, family, genus, and zOTU) throughout the experiment (Figure 2). At DAH 7, larvae nursed on CF showed higher observed species richness and greater bacterial diversity than larvae nursed on SFW (Figure 3). These differences persisted after the diet shift, resulting in distinct diversity patterns among the diet treatments. Larvae maintained on CF throughout both stages (CF to CF) consistently exhibited the highest bacterial diversity, whereas larvae kept on SFW throughout (SFW to SFW) showed the lowest diversity. In larvae that switched from CF to SFW (CF to SFW), species diversity declined progressively after the shift at DAH 7. In contrast, larvae that transitioned from SFW to CF (SFW to CF) exhibited an increase in diversity after the switch.

**Figure 4:**
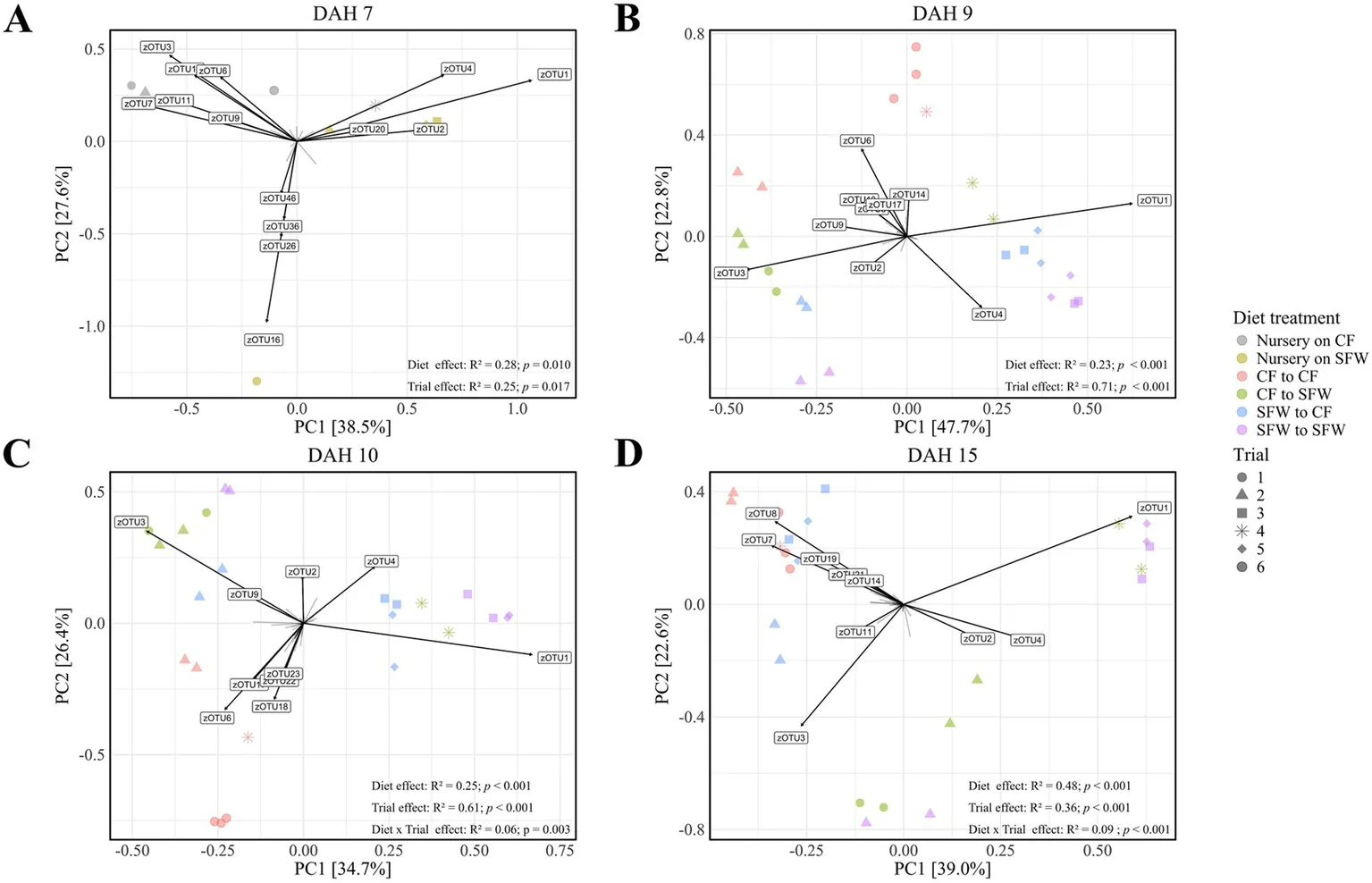
Redundancy analysis (RDA) plots showing clustering of black soldier fly larval samples based on Hellinger-transformed relative abundance data. Plots show clustering of samples at **(A)** days after harvest (DAH) 7 on nursery substrates chicken feed (CF) and artificial supermarket food waste (SFW) and at **(B)** DAH 9, **(C)** 10 and **(D)** 15 after the diet shift from nursery substrates (CF or SFW) to fattening substrates (CF or SFW). Effects of diet treatment, trial, and their interaction on microbial community composition were assessed using PERMANOVA based on Bray-Curtis dissimilarities calculated from Hellinger-transformed relative abundance data and are reported as explained variation (R^2^) and significance levels (*p*-values).

Beta-diversity analysis (PERMANOVA) of bacterial community composition (Figure 4) showed no significant effects of nursery diet (*p* = 0.231, R^2^ = 0.28) or experimental trial (*p* = 0.755, R^2^ = 0.56) at DAH 7. However, the limited replication at DAH 7, with at most one biological replicate per nursery diet within each trial, may have reduced the statistical power to detect subtle treatment effects. Following the shift from nursery to fattening substrates, significant effects of both diet treatment and experimental trial emerged. Significant differences among the four diet treatments were detected at DAH 9 (*p* < 0.001, R^2^ = 0.23), DAH 10 (*p* < 0.001, R^2^ = 0.25) and DAH 15 (*p* < 0.001, R^2^ = 0.48). Furthermore, differences between trials were identified across all later sampling days (*p* < 0.001, R^2^ = 0.71 for DAH 9; *p* < 0.001, R^2^ = 0.61 for DAH 10; *p* < 0.001, R^2^ = 0.36 for DAH 15). At DAH 9 and DAH 10, variation between trials accounted for a larger proportion of the observed microbial community variation than diet treatment, indicating a substantial contribution of trial-specific effects. In addition, a significant diet treatment-by-trial interaction at DAH 10 (*p* = 0.003, R^2^ = 0.06) and DAH 15 (*p* < 0.001, R^2^ = 0.09) showed that the microbial response to the diet shift differed among experimental trials.

To assess the persistence of nursery substrate effects following the diet shift, separate analyses were conducted within each fattening treatment (Table 5). For larvae fattened on CF, the proportion of variation explained by nursery substrate remained stable between DAH 9 (R^2^ = 0.45) and DAH 10 (R^2^ = 0.46) and decreased only at DAH 15 (R^2^ = 0.21). In larvae fattened on SFW, the nursery substrate explained a large proportion of the variation at DAH 9 (R^2^ = 0.47) but had a less pronounced effect on DAH 10 (R^2^ = 0.28) and DAH 15 (R^2^ = 0.15). Differences between experimental trials accounted for the largest proportion of variation across all sampling days in both fattening treatments.

**Table 5:** PERMANOVA analysis of the effects of nursery diet and trial on larval gut bacterial community composition at days after harvest (DAH) 9, 10, and 15, for each fattening substrate (chicken feed (CF) and artificial supermarket food waste (SFW)). Larvae were nursed on either CF or SFW prior to transfer to the fattening substrate. Analyses were based on Bray-Curtis dissimilarities calculated from Hellinger-transformed relative abundance data and are reported as explained variation (R^2^) and significance levels (*p*-values).

Fattening substrate	DAH	Effect of nursery substrate	Effect of trial
CF	9	R^2^ = 0.45 (*p* = 0.0001)	R^2^ = 0.48 (*p* = 0.0001)
CF	10	R^2^ = 0.46 (*p* = 0.0001)	R^2^ = 0.49 (*p* = 0.0001)
CF	15	R^2^ = 0.21 (*p* = 0.0001)	R^2^ = 0.60 (*p* = 0.0001)
SFW	9	R^2^ = 0.47 (*p* = 0.0001)	R^2^ = 0.50 (*p* = 0.0004)
SFW	10	R^2^ = 0.28 (*p* = 0.0023)	R^2^ = 0.56 (*p* = 0.0012)
SFW	15	R^2^ = 0.15 (*p* = 0.0017)	R^2^ = 0.77 (*p* = 0.0002)

**Figure 5:**
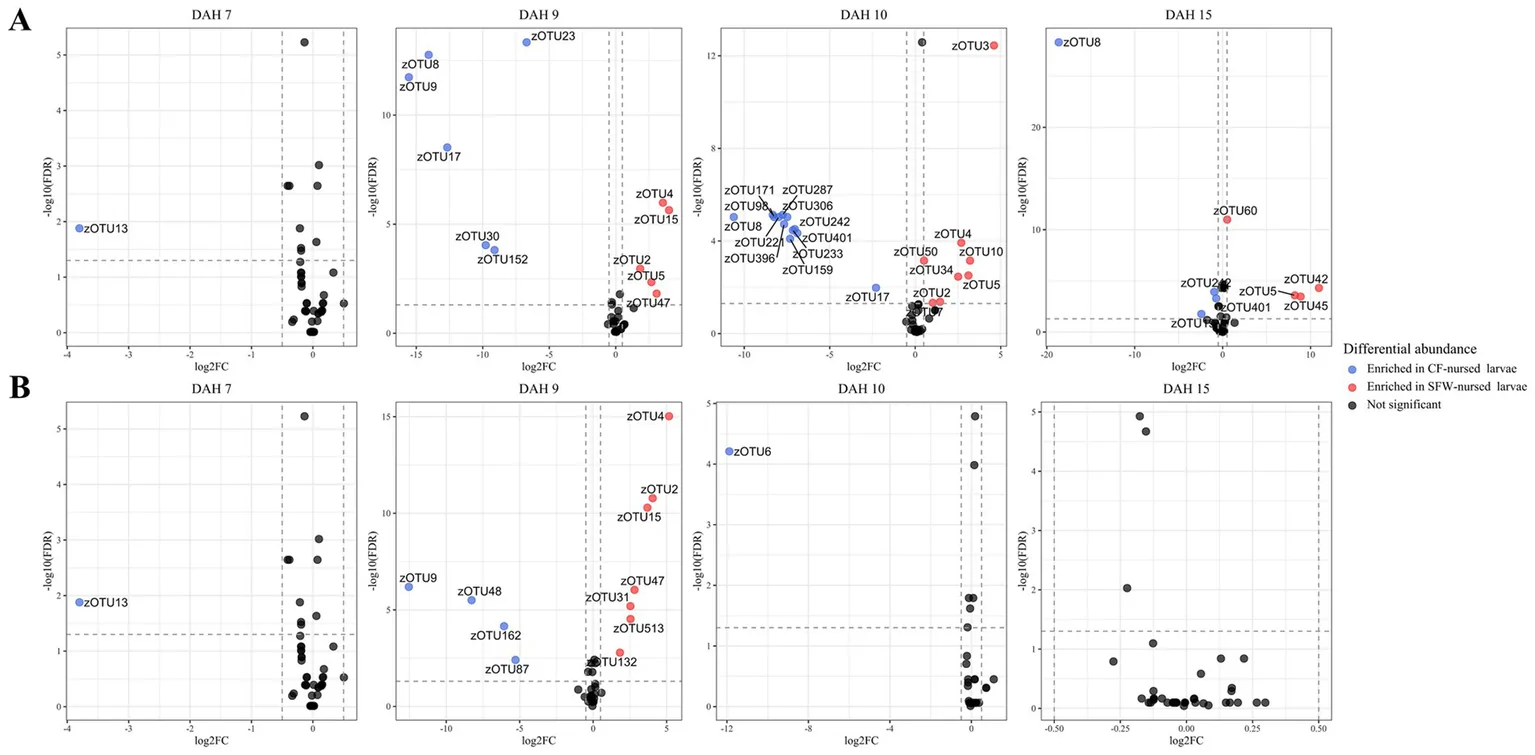
Volcano plots showing biomarkers of the black soldier fly larval gut bacterial community. The colored dots indicate biomarkers identified in larvae reared on the nursery diets Chicken Feed (CF, blue dot) and Artificial Supermarket Food Waste (SFW, red dot) and are shown at days after harvest (DAH) 7, 9, 10 and 15 for larvae fattened on **(A)** CF and **(B)** SFW. Taxa were considered significantly different between nursery diets when the adjusted *p* < 0.05 (following Benjamini-Hochberg correction) and at a log2foldchange larger than 0.5.

After correcting for inter-trial differences, the DESeq2 differential abundance analysis further revealed treatment-dependent differences in microbiome composition (Figure 5). At DAH 7, only one biomarker was significantly enriched (zOTU13) in CF-nursed larvae compared to SFW-nursed larvae, indicating that only limited differences in individual biomarkers could be detected between the nursery treatments at the end of the nursery phase. It should be noted, however, that the relatively low number of biological replicates and variation among experimental trials may have limited the statistical power of the DESeq2 analysis, potentially obscuring treatment-related differences in community composition. Following transfer to the fattening substrate, nursery-associated differences persisted but showed distinct temporal patterns depending on the fattening substrate. For larvae fattened on CF, six zOTUs were significantly enriched in CF-nursed larvae and five in SFW-nursed larvae at DAH 9. The number of biomarkers increased at DAH 10, with twelve and eight zOTUs enriched in CF- and SFW-nursed larvae, respectively. By DAH 15, four zOTUs remained significantly enriched in each nursery treatment. In larvae fattened on SFW, four zOTUs were significantly enriched in CF-nursed larvae and seven in SFW-nursed larvae at DAH 9. At DAH 10, only a single zOTU remained enriched in CF-nursed larvae, while no zOTUs were enriched in SFW-nursed larvae. At DAH 15, no significant biomarkers associated with nursery treatment were detected in SFW-fattened larvae.

## 4 Discussion

### 4.1 Larval growth responses to dietary transition

#### 4.1.1 Early growth dynamics before and after dietary transition

The choice of nursery substrate clearly influenced larval growth during the first days of the fattening stage. Although no significant difference in larval weight between CF- and SFW-nursed larvae was detected at the start of the fattening phase (DAH 7), a possible effect of the nursery diet on larval performance at this time point might be constrained by the limited number of replicates (*n* = 4 for CF and *n* = 3 for SFW) and by a substantial trial effect on larval weight. Specifically, in trial 2, larvae from both nursery substrates exhibited substantially reduced body weights compared to the other trials, indicating that small variations between experimental trials can influence larval growth.

At DAH 8 and 9, however, a clear effect of nursery substrate occurred. Across both fattening substrates, larvae nursed on CF achieved higher individual body weights compared with those nursed on SFW. This indicates that the conditions provided by CF during the nursery period resulted in an early growth advantage that persisted during the fattening stage. Two factors likely contributed to this effect: first, CF had a more favorable nutritional profile than SFW, containing higher levels of energy, carbohydrates, protein, and fat. Second, the WHC of CF was substantially higher, resulting in lower proportions of free water in the substrate. High free-water content is known to disrupt BSFL feeding behavior: overly wet substrates are typically avoided, reducing feed intake and increasing the energetic cost of searching for more suitable microhabitats (Bellezza Oddon et al., 2024). This combination of lower nutrient density and higher free-water levels likely explains the reduced early growth observed in SFW-nursed larvae. These findings are consistent with Vandeweyer et al. (2023), who also reported diminished larval performance on SFW and attributed it to the combined effects of poor nutritional quality and elevated free-water content.

Despite this initial disadvantage, SFW-nursed larvae eventually reached individual body weights comparable to those of CF-nursed larvae, although convergence occurred earlier under SFW fattening (by DAH 10) than under CF fattening (between DAH 10 and 14). Several factors may explain these differences in convergence timing. First, the observed pattern may suggest that larvae adjust more rapidly when the fattening substrate matches that used during the nursery stage. A second possibility is that convergence occurred more rapidly under SFW fattening because this substrate supported lower overall larval growth. As larvae attained lower final body weights on SFW, the initial weight difference between CF- and SFW-nursed larvae may have been compressed more rapidly, resulting in earlier convergence. Alternatively, the variation in convergence timing might be related to larval survival. At DAH 15, survival of SFW-nursed larvae was lower than that of CF-nursed larvae under both fattening diets. Although survival was not assessed at earlier time points, lower survival may already have been present during part of the fattening phase, reducing larval density and feed competition (Frooninckx et al., 2024) and thereby contributing to the observed convergence in body weight. Finally, differences in substrate-associated microbiota and the extent of microbiome adaptation required following dietary transition may also have influenced convergence dynamics (see Section 4.2). Future studies are needed to determine the mechanisms underlying these differences in convergence timing.

#### 4.1.2 Final performance outcomes

Despite the convergence in individual body weight, differences in final performance remained evident. Within the CF fattening substrate, SFW-nursed larvae reached a significantly higher individual weight at DAH 15, whereas CF-nursed larvae exhibited a decline in weight between DAH 14 and 15. This may indicate that nursery on SFW delayed the onset of prepupal weight loss, potentially allowing larvae to attain equal or greater final weights at a later developmental stage. However, as developmental stage was not assessed morphologically, this interpretation remains speculative. Notably, the higher individual weights of SFW-nursed larvae were accompanied by lower survival rates, suggesting a potential trade-off between growth and viability. Consequently, total larval yield did not differ significantly between nursery treatments. In contrast, within the SFW fattening substrate, individual weight at DAH 15 was unaffected by nursery diet, but the higher survival of CF-nursed larvae resulted in a greater total larval weight.

Within both fattening substrates, CF-nursed larvae consistently exhibited higher survival than SFW-nursed larvae. This may partly reflect the lower body weight of SFW-nursed larvae at the start of the fattening phase. Smaller larvae may have been less capable of coping with either the transition to CF or the continued exposure to the high free-water content of SFW, potentially increasing mortality risk (Frooninckx et al., 2024). However, because the timing of mortality was not recorded, the mechanisms underlying these survival differences remain uncertain.

Regardless of nursery diet, larvae performed better when fattened on CF. At DAH 15, individual body weight, survival, and total larval weight were all higher under CF fattening, consistent with the more favorable nutritional and physical characteristics of this substrate (Section 4.1.1). However, larvae fattened on SFW, as well as larvae transitioned from SFW nursery to CF fattening, did not exhibit the decline in body weight typically associated with the onset of the prepupal stage. As developmental stage was not assessed morphologically, treatment differences may partly reflect variation in developmental progression rather than true differences in growth performance.

### 4.2 Impact of diet shift on microbiome

In addition to differences in nutritional composition, other factors may have contributed to the growth patterns observed following the diet shift. Changes in substrate composition have been associated with shifts in the larval microbiome (Eke et al., 2023; Vandeweyer et al., 2023), which are hypothesized to enable the utilization of resources available in the new diet (IJdema et al., 2025). Hence, if microbiome restructuring is required after a dietary change, an adjustment period may be necessary before larvae can fully exploit the new nutritional potential of the new substrate, which in turn can influence larval growth. In addition, the significant trial effect observed in this study suggests that factors varying between experimental runs also influenced larval performance. Therefore, growth responses following dietary transition should be interpreted as the result of multiple interacting factors rather than diet composition alone.

#### 4.2.1 Microbial community convergence and trial-dependent variation following dietary transition

At the end of the nursery stage (DAH 7), microbiome differences between CF- and SFW-nursed larvae were limited, with only a single differentially abundant biomarker detected between the two nursery treatments. Although differences in relative abundance profiles were observed in the compositional barplots, these were not reflected in the differential abundance analysis. This discrepancy may be partly attributable to the limited number of biological replicates at DAH 7 (*n* = 4 for CF and *n* = 3 for SFW), with at most one biological replicate per nursery diet within each trial, which likely reduced the statistical power to detect treatment-associated differences.

Following the dietary transition, microbiome composition gradually shifted from being primarily associated with the nursery substrate toward being increasingly shaped by the fattening substrate. However, the rate of this transition differed depending on the direction of the diet shift. At DAH 9, CF- and SFW-nursed larvae had various biomarkers on both fattening substrates, indicating a microbial shift after the transition toward a new fattening substrate. From DAH 10 onwards, larvae fattened on SFW exhibited fewer differentially abundant biomarkers between CF- and SFW-nursed individuals than larvae fattened on CF. This pattern suggests that the microbial signature associated with the nursery substrate disappeared more rapidly under SFW fattening, resulting in an earlier convergence toward a microbiome characteristic of the SFW diet. In contrast, larvae fattened on CF retained a stronger legacy effect of the nursery substrate, as reflected by the larger number of differentially abundant biomarkers persisting throughout the fattening period.

These different convergence dynamics may be related to differences in microbiome complexity associated with the respective diets. Although the microbial communities of the substrates themselves were not characterized in the present study, previous work reported lower microbiome diversity in SFW than in CF (Vandeweyer et al., 2023). Larvae continuously reared on SFW maintained a comparatively low-diversity microbiome that, from DAH 9 onwards, was dominated by only five zOTUs, three of which (zOTU2, zOTU4, and zOTU6) belonged to the family *Lactobacillaceae* (Figure 2). In contrast, larvae continuously reared on CF harboured a more diverse bacterial community, including several zOTUs uniquely associated with this substrate. Given this lower microbiome diversity and dominance of a limited number of taxa under SFW rearing, a transition toward the SFW-associated microbiome may require the establishment of only a limited number of dominant taxa, whereas convergence toward the more complex CF-associated community may require a more extensive restructuring of the microbiome and therefore occur more gradually.

Notably, the timing of these microbiome dynamics coincided with differences in larval growth responses, as SFW-nursed larvae attained body weights comparable to those of CF-nursed larvae earlier under SFW fattening than under CF fattening (Section 4.1). Although the present study does not allow causal relationships between microbiome composition and growth performance to be established, the correspondence between the timing of microbiome convergence and weight convergence suggests that microbiome adaptation may contribute to the response of larvae to dietary transition. Further research is needed to evaluate the extent to which microbial community dynamics influence growth following a change in diet.

Despite these differences in convergence dynamics, no nursery-specific biomarkers were consistently detected across all sampling points. However, the absence of persistent biomarkers should be interpreted with caution, as substantial inter-trial variation and the limited number of biological replicates may have reduced the statistical power to detect subtle but biologically relevant differences, particularly at DAH 7.

Variation between trials, as reflected by the clustering patterns in the RDA plots, contributed substantially to the observed microbiome patterns. At DAH 9 and DAH 10, trial effects explained approximately threefold and twofold more variation, respectively, than diet treatment, indicating that factors varying among experimental runs strongly influenced microbiome composition during the early fattening phase. Such between-trial variability may reflect stochastic processes during early microbiome assembly, where random colonization events and priority effects can lead to divergent community trajectories even under similar conditions, and should therefore be considered when interpreting microbial community data (Hayashi et al., 2026). The presence of these trial effects highlights the importance of accounting for between-trial variability when evaluating microbiome responses to dietary transitions. Future studies should therefore include a greater number of biological replicates within individual trials to enable a more robust separation of treatment and trial effects.

#### 4.2.2 Microbial biomarkers associated with dietary transition

Most biomarkers identified after the dietary transition belonged to the family *Lactobacillaceae*, particularly members of the genus *Lactobacillus*. These taxa were detected in both CF- and SFW-nursed larvae, suggesting that dietary transitions primarily affected the relative abundance of closely related bacterial groups rather than inducing major shifts in dominant microbial families. This pattern may reflect niche differentiation among taxa adapted to specific substrate characteristics. Interestingly, the genus *Lactobacillus* has previously been associated with artificial diets and dietary transitions in silkworms, suggesting a potential involvement in microbiome adaptation to dietary change (Lei et al., 2024). Although *Lactobacillaceae* accounted for the majority of detected biomarkers, a limited number of biomarkers affiliated with other bacterial families were also identified and may provide additional insight into microbiome responses following dietary transition.

At DAH10, two *Pediococcus* species (zOTU10 and zOTU34) were enriched in SFW-nursed larvae fattened on CF. *Pediococcus* has previously been found in CF-fed BSFL in other studies (Schreven et al., 2022), although its functional role in BSF larvae remains unknown. In other insect species, however, probiotic supplementation of *Pediococcus pentosaceus* strain improved survival, growth performance and feed efficiency in silkworm (*Bombyx mori*) larvae (Zeng et al., 2024), while a host-associated *P. pentosaceus* strain promoted growth and survival in *Tenebrio molitor* larvae (Lecocq et al., 2021). In the present study, higher body weights of SFW-nursed larvae became apparent only after DAH 10. However, lower survival and therefore potentially lower feed competition in these treatments may also have contributed to the observed performance differences. Since no microbiome samples were collected between DAH 10 and DAH 14, no direct relationship between *Pediococcus* abundance and larval growth can be established. Nevertheless, its potential contribution to the observed performance differences cannot be excluded and warrants further investigation.

Two members of the family *Leuconostocaceae*, *Weissella* (zOTU6) and an unidentified *Leuconostocaceae* sp. (zOTU48), were enriched in CF-nursed larvae fattened on SFW at DAH 9 and 10. *Weissella* has previously been reported in BSFL reared on CF and is generally most abundant during early larval stages before declining with age (Gorrens et al., 2022; Schreven et al., 2022; Vandeweyer et al., 2023). Its enrichment shortly after the dietary transition therefore suggests persistence of nursery-associated microbiome differences during the early fattening phase. Although its role in BSFL remains unclear, *Weissella* was enriched in silkworms fed artificial diets and was highlighted as a potential probiotic candidate in the context of dietary adaptation (Lei et al., 2024).

*Enterococcus* (zOTU8) was enriched in CF-nursed larvae fattened on CF on DAH 9, 10, and 15 relative to SFW-nursed larvae reared on the same fattening substrate. This genus is commonly associated with BSFL, has been consistently detected in studies investigating the larval bacterial community (IJdema et al., 2022) and has previously been reported in late-stage BSFL microbiomes (Vandeweyer et al., 2023). Interestingly, this enrichment was not observed in SFW-nursed larvae fattened on the same CF substrate, suggesting that nursery-associated differences in *Enterococcus* abundance persisted despite exposure to an identical fattening diet. Although its functional significance in BSFL remains incompletely understood, *Enterococcus* has been associated with increased feeding behavior (Yang et al., 2021) and positively correlated with lipase activity (Chen et al., 2023), suggesting a potential role in nutrient utilization.

Particular caution is warranted when interpreting *Enterobacteriaceae*-associated biomarkers. Members of this family were identified across all four treatment groups, suggesting that *Enterobacteriaceae* constitute an important and widespread component of the BSFL microbiome. However, RDA analyses indicated that *Enterobacteriaceae*-related taxa also contributed substantially to the observed trial effects. In particular, differences in the relative abundance of *Providencia* (zOTU1) and an unidentified *Enterobacteriaceae* taxon (zOTU3) appeared to drive much of the variation observed among trials. While zOTU3 was predominantly associated with trials 1 and 2, zOTU1 was mainly observed in the remaining trials, suggesting potential niche differentiation or competitive interactions between these taxa. As both taxa belong to the *Enterobacteriaceae* family, their distribution patterns may reflect differences in community assembly between trials rather than consistent responses to dietary treatment. Moreover, *Enterobacteriaceae* comprise a highly diverse bacterial family with members exhibiting a broad range of ecological functions, making it difficult to infer biological significance from family-level associations alone. Further studies providing species-level identification and functional characterization are therefore needed to clarify their role in BSFL microbiome dynamics following dietary transitions.

## 5 Conclusion

This study demonstrates that a diet shift affects larval growth during the early phase after the diet transition and has lasting effects on the microbiome. During the first days following the transition to the fattening substrate, larvae nursed on CF consistently achieved higher body weights than larvae nursed on SFW, regardless of the fattening substrate. However, the effect of nursery diet on growth was transient, and body weights gradually converged over time. The timing of this convergence depended on the fattening substrate, occurring earlier under SFW fattening than under CF fattening. Interpretation of these growth responses is complicated by the consistently lower survival of SFW-nursed larvae, making it difficult to determine the relative contributions of dietary transition and density-dependent effects. Microbiome analyses revealed that both the nursery and fattening substrates influenced bacterial community composition, although the persistence of nursery diet effects depended on the fattening substrate. Furthermore, substantial trial effects contributed to microbiome variation, highlighting the importance of stochastic processes during microbiome assembly. Although causality could not be established, the temporal correspondence between microbiome dynamics and growth responses suggests a role for microbial community adaptation in larval responses following dietary transitions. Together, these results highlight that both deterministic factors, such as diet history, and stochastic processes influence microbiome assembly during BSFL development.

## Data Availability

The datasets presented in this study can be found in online repositories. The names of the repository/repositories and accession number(s) can be found at: https://www.ebi.ac.uk/ena, PRJEB114381.
