# Supplementary material for: Impact of nursery-to-fattening dietary transitions on gut microbiome composition and growth performance in black soldier fly larvae
Source: Front Microbiol. 2026 Aug 19;17:1901348. doi: 10.3389/fmicb.2026.1901348 (PMC13534125; doi:10.3389/fmicb.2026.1901348)
Supplement: Supplementary file 1 [file Data_Sheet_1.PDF]

Supplementary Material

1 Supplementary Data

1.1 Supplementary Tables

Supplementary Table 1. Number of replicates for each of the four diet treatments (CF to CF, CF to SFW, SFW to SFW and SFW to CF; CF: chicken feed, SFW: supermarket food waste) across six different trials.

	Diet treatment			
**Trial**	**CF to CF**	**CF to SW**	**SW to SW**	**SW to CF**
**1**		2		
**2**	2	2	2	2
**3**			2	2
**4**	1	2		
**5**			2	2
**6**	3			

Supplementary Table 2. Overview of the supplementary datasets used for microbiome analyses, including filtered read counts per sample, zOTU taxonomic classifications, and sample metadata.
